# Relationship between PPAR‐γ gene polymorphisms and ischemic stroke risk: A meta‐analysis

**DOI:** 10.1002/brb3.2434

**Published:** 2021-11-10

**Authors:** Fan Cheng, Xiao‐Min Si, Gong‐Li Yang, Lan Zhou

**Affiliations:** ^1^ Center of Cardiopulmonary Rehabilitation Taihe Hospital Hubei University of Medicine Shiyan China; ^2^ Department of Neurology Taihe Hospital Hubei Key Laboratory of Embryonic Stem Cell Research Hubei University of Medicine Shiyan China; ^3^ Department of Gastroenterology Shenzhen University General Hospital Shenzhen University Shenzhen China

**Keywords:** meta‐analysis, polymorphism, PPAR‐γ, stroke

## Abstract

**Background:**

Published researches have suggested some associations between PPAR‐γ and ischemic stroke (IS) development. This meta‐analysis was conducted to evaluate the association between PPAR‐γ gene polymorphisms and IS risk.

**Materials and methods:**

A systematic search was conducted in PubMed, Embase, Web of Science, China National Knowledge Infrastructure, and WanFang databases. The pooled association of odd ratios (ORs) and its 95% confidence interval (CI) was calculated to assess the IS risk of PPAR‐γ rs1801282 C/G and rs3856806 C/T polymorphisms. Furthermore, the heterogeneity test, cumulative analyses, sensitivity analyses, and publication bias were conducted.

**Result:**

Sixteen publications with 3786 cases and 5343 controls were identified. Overall findings indicated that rs1801282 C/G polymorphism may be associated with an increased risk for IS (GG vs. CC: OR = 2.17 95%CI = 1.09–4.35, *p* = .03, *I* ^2 ^= 0%; GG vs. CC+CG: OR = 2.15, 95%CI = 1.07–4.32, *p* = .03, *I* ^2 ^= 0%). The similar results were also found in the subgroup analysis. In addition, no significant association was observed between rs3856806 C/T polymorphism and IS risk.

**Conclusion:**

In conclusion, our study showed that PPAR‐γ rs1801282 C/G polymorphism probably plays an important role in IS occurrence. The result should be verified with more studies in the future.

## Graphical abstract

Ischemic strokes (IS) are one of the most common diseases resulting in disabilities and death among adults. Our meta‐analysis indicated that the individuals with G mutation of rs1801282 C/G polymorphism would face an increased risk of IS. But there was no significant association between rs3856806 C/T polymorphism and IS risk.

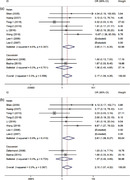

## 1 INTRODUCTION

Stroke is one of the most common medical events resulting in disability and death among adults. More than 13.7 million new stroke cases and 5.5 million deaths, in addition to 116.4 million disability‐adjusted life years, were recorded worldwide in 2016 alone (Collaborators GBDS, 2019). Ischemic stroke (IS) is considered an atherosclerotic and thromboembolic event that results in decreased blood flow to the brain tissue, leading to subsequent infarction (Feigin et al., 2009). With an increase in the elderly population, the incidence of IS is projected to exhibit a sharp upsurge in the near future. IS, one of the most prevalent cerebrovascular events, has turned into a tragedy for affected individuals, as it brings heavy mental and financial burden for the family and society. This has resulted in a significant decline in the quality of life of patients in both developed and developing countries. Etiology and pathogenesis studies have been extensively conducted on IS; however, the underlying mechanism remains unclear. Cerebrovascular atherosclerosis and secondary ischemic brain injury are the most common risk factors that ultimately result in cerebral thrombosis and IS. Moreover, other factors such as environmental pollution, hypertension, genetic background, and unhealthy lifestyle habits have additionally been suggested to be associated with IS development. Increasing evidence suggests the existence of a strong polygenic inheritance factor for IS etiology, including abnormal gene expression and genetic mutations.

Peroxisome proliferator‐activated receptor‐γ (PPAR‐γ) belongs to the nuclear receptor superfamily, which mainly mediates ligand‐dependent transcriptional activation and repression. PPAR‐γ plays a key role in the regulation of adipocyte differentiation, lipid metabolism, and insulin sensitivity in vivo, with PPAR‐γ2 exhibiting a higher activity in adipocytes than other types. Accumulating evidence indicates that PPAR‐γ participates in the regulation of adipocyte differentiation, lipid metabolism, and the concentration of lipids and cholesterol. Dyslipidemia is a well‐established risk factor for cardiovascular diseases, which triggers the development of atherosclerosis and increases the risk of IS risk. PPAR‐γ also plays an important role in regulating inflammatory processes, participates in vascular endothelial cell repair, and inhibits thrombosis formation (D. Li et al., 2005). A recent study demonstrated that PPAR‐γ agonists exert an active anti‐inflammatory effect in IS models and protect against thrombosis by downregulating the activation of nuclear factor‐κB and decreasing the expression of proinflammatory cell adhesion molecules (Jin et al., 2015).

The PPAR‐γ gene is located on chromosome 3p25, is comprised of 100Kb DNA bases, and consists of nine exons. The rs1801282 C/G and rs3856806 C/T polymorphisms are the most common single nucleotide polymorphism (SNP) loci in the PPAR‐γ gene, and have been extensively studied in different ethnic groups and found to be associated with the risk of several metabolic diseases, including diabetes and its complications. This mutation may change the transcriptional activity and subsequently alter the protein synthesis of PPAR‐γ (Masugi et al., 2000). This leads to abnormal lipid metabolism and subcellular metabolism in arterial foam cells, resulting in the formation of atherosclerosis and an altered risk of stroke (Grbić et al., 2018). Furthermore, PPAR‐γ agonists have been extensively studied as potential neuroprotective agents in recent decades (Kinouchi et al., 2018; Y. Li et al., 2019; Shimazu et al., 2005).

In 2005, Shen and Ha (2005) conducted the first case–control study and found no significant association between the rs1801282 C/G polymorphism and IS risk in a Chinese population. However, study results that followed Shen et al. on genetic associations of this SNP with IS risk have been controversial and inconclusive. Therefore, this meta‐analysis was conducted to amalgamate the results of existing studies in order to elucidate the association of rs1801282 C/G and rs3856806 C/T polymorphisms with IS risk.

## 2 MATERIALS AND METHODS

This meta‐analysis was conducted according to the online guidance of the Preferred Reporting Items for Systematic Reviews and Meta‐Analyses (PRISMA) statement. All collected data were extracted from published studies, and no ethical issue was involved (Moher et al., 2009).

### 2.1 Literature search

The PubMed, Embase, Web of Science, China National Knowledge Infrastructure, and WanFang databases were explored online, focusing on the association between PPAR‐γ polymorphisms and IS susceptibility before April 1, 2021. Research articles published only in English and Chinese were selected for this study. The bibliographies of relevant articles were retrieved for potential studies. The strategy was listed as follows (e.g., in PubMed):

#1 Peroxisome proliferator‐activated receptor‐γ
#2 PPAR‐γ
#3 PPARG
#4 #1 or #2 or #3
#5 rs1801282
#6 rs3856806
#7 Pro12Ala
#8 polymorphism
#9 Variant
#10 Mutation
#11 #5 or #6 or #7 or #8 or #9 or #10
#12 Ischemic stroke
#13 Stroke
#14 #12 or #13
#15 #4 and #11 and #14

### 2.2 Inclusion and exclusion criteria

The criteria for inclusion of identified studies for the purpose of this meta‐analysis were as follows: (1) case–control studies on the association between PPAR‐γ rs1801282 C/G, rs3856806 C/T polymorphisms, and IS risk; (2) studies supplying sufficient information on the genotypes in both case and control groups with respect to evaluate the odd ratios (ORs) and 95% confidence intervals (CIs); (3) studies published either in English or Chinese; (4) the polymorphism locus was calculated with at least three studies; and the largest or latest data with more adequate information were collected when duplicate publications or overlapping data were presented. The exclusion criteria were as follows: (1) review articles, case reports, and animal experiments; (2) biological fundamental research; (3) studies without sufficient data; and (4) duplicate or overlapping data on the same theme.

### 2.3 Data extraction and quality evaluation

Two authors (Cheng and Zhou) independently reviewed the included studies and extracted the relevant information: the first author’ name, year of publication, study country, ethnicity difference, control design, genotyping method, sample sizes of the cases and controls, frequency of the genotype distribution in the cases and control groups, the degree of Hardy–Weinberg equilibrium (HWE) in the control group, minor allele frequency, and Newcastle–Ottawa scale (NOS) evaluation. The NOS was adopted to evaluate the quality of the included studies. The scores ranged from 0 (worst) to 11 (best) (Table 1). Studies with a score of eight points or higher indicate a good research quality.

**TABLE 1:** Characteristics of case–control studies on peroxisome proliferator‐activated receptor‐γ (PPAR‐γ) polymorphisms and ischemic stroke risk

								Genotype distribution									
First author	Year	Country	Ethnicity	Control design	Genotype method	Case	Control	Case			Control			*p* for HWE	MAF	NOS evaluation	Matching criteria
Rs1801282								CC	CG	GG	CC	CG	GG				
Shen	2005	China	Asian	PB	PCR‐RFLP	70	95	66	3	1	89	6	0	.75	0.03	9	Healthy check‐ups
Lee‐1	2006	Korea	Asian	HB	PCR‐RFLP	302	424	290	12	0	384	40	0	.31	0.05	10	Age‐matched health and diabetes controls
Lee‐2	2007	Korea	Asian	PB	PCR‐RFLP	134	129	128	6	0	117	12	0	.58	0.05	10	Health check‐ups
Huang	2007	China	Asian	PB	PCR‐RFLP	199	200	171	18	1	177	23	0	.39	0.06	9	Healthy check‐ups
Zafarmand	2008	Netherlands	Caucasian	PB	Multilocus genotyping assay	49	1519	38	10	1	1143	346	30	.52	0.13	10	NA
Bazina	2015	Croatia	Caucasian	PB	PCR‐RFLP	114	187	84	27	3	140	44	3	.83	0.13	9	Sex and age matched healthy staff
Tong‐1	2015	China	Asian	PB	TaqMan	100	100	80	19	1	78	19	3	.19	0.13	9	Age and gender and ethnically matched normal healthy controls
Tong‐2	2016	China	Asian	PB	TaqMan	648	648	606	40	2	580	67	1	.51	0.05	10	Age and gender and ethnically matched normal healthy controls
Li	2016	China	Asian	HB	PCR‐RFLP	302	272	274	26	2	250	21	1	.44	0.04	9	NA
Wang	2019	China	Asian	PB	SNaPshot Multiplex Kit	895	883	756	129	10	807	75	1	.59	0.04	10	Age‐ and sex‐matched healthy controls
Rs3856806								CC	CT	TT	CC	CT	TT				
Yuan	2008	China	Asian	PB	PCR‐RFLP	293	203	150	134	14	126	72	5	.15	0.20	9	NA
Lu	2009	China	Asian	PB	PCR‐RFLP	114	120	87	23	4	72	41	7	.72	0.23	9	Healthy check‐ups
Liu	2010	China	Asian	PB	PCR‐RFLP	168	165	128	36	4	100	60	5	.26	0.21	8	Healthy check‐ups
Sun	2010	China	Asian	PB	PCR‐RFLP	90	94	45	42	3	56	36	2	.16	0.21	9	Age‐ and sex‐matched healthy check‐ups
Wei	2013	China	Asian	PB	PCR‐RFLP	112	112	87	19	6	78	25	9	<.01	0.19	8	Healthy check‐ups
Chehaibi	2014	Tunisia	Caucasian	PB	PCR‐RFLP	196	192	143	39	14	118	46	28	<.01	0.27	8	Individuals with normal glucose
Wang	2019	China	Asian	PB	SNaPshot Multiplex Kit	895	883	515	322	58	587	274	22	.13	0.18	10	Age‐ and sex‐matched healthy controls

*Note*: Hardy–Weinberg equilibrium (HWE) in control.

Abbreviations: HB, hospital‐based control; NA, not available; NOS, Newcastle–Ottawa scale; PB, population‐based control. PCR‐RFLP; polymerase chain reaction–restriction fragment length polymorphism.

### 2.4 Statistical analysis

The ORs and 95% CIs were calculated to examine the statistical power between PPAR‐γ polymorphisms and IS risk using five genetic models: allelic model (rs1801282: G vs. C; rs3856806: T vs. C), codominant models: heterozygous model (rs1801282: CG vs. CC; rs3856806: CT vs. CC) and homozygous model (rs1801282: GG vs. CC; rs3856806: TT vs. CC), dominant model (rs1801282: CG+GG vs. CC; rs3856806: CT+TT vs. CC), and recessive model (rs1801282: GG vs. CC+CG; rs3856806: TT vs. CC+CT). Heterogeneity among the included studies was examined using Cochran's *Q* and *I* ^2^ tests. A fixed‐effects model was adopted when *I* ^2^ was ≤40%; otherwise, a random‐effects model was adopted. Subgroup analyses were conducted based on ethnic differences, control design, number of subjects, and the HWE status. Subgroup analysis was conducted only if data from at least two studies were available. Meta‐regression analysis was performed to identify the potential factors that may have contributed to the heterogeneity. A cumulative meta‐analysis was conducted to determine the change in tendencies in the results. Sensitivity analysis was used to examine the stability of the results by removing each study step by step. Publication biases were assessed using Egger's linear regression test and Begg's funnel plots. All statistical analyses were performed using the STATA version 14.0 (Stata Corporation, College Station, TX, USA). Statistical significance was set at *p* <.05.

## 3 RESULTS

### 3.1 Study characteristics

**FIGURE 1:**
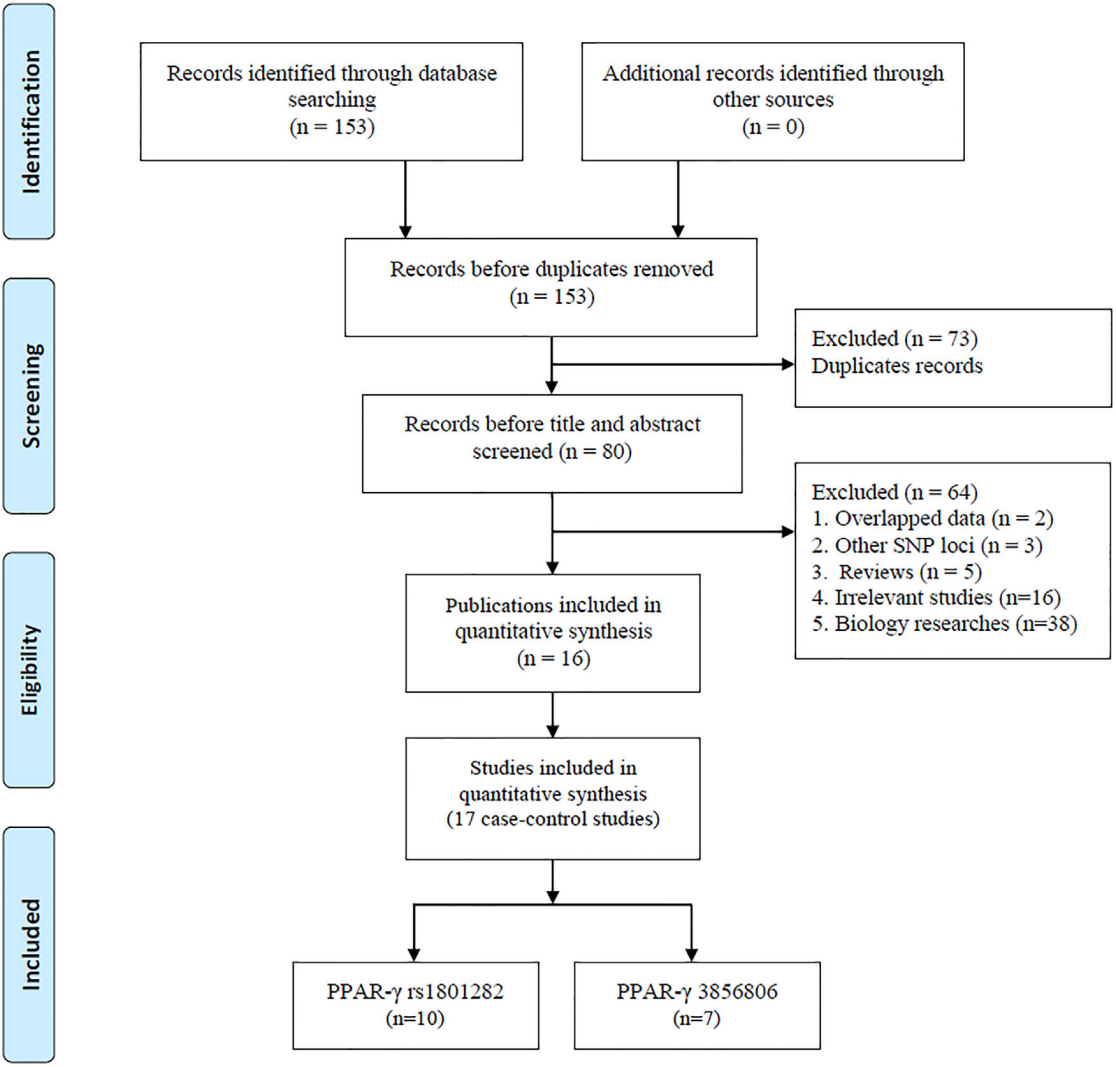
Flow diagram of the study selection process

According to the search strategy utilizing a variety of literature retrieval databases, a total of 153 studies were initially identified, of which 73 were excluded for duplications through title and abstract screening. After the full‐text review, 64 studies were excluded further (overlapping data [*n* = 2], other polymorphism loci [*n* = 3], reviews [*n* = 3], irrelevant studies [*n* = 16], and biology studies [*n* = 38]) (Figure 1). Finally, 16 studies involving 3786 patients and 5343 controls were included in this meta‐analysis (Bazina et al., 2015; Chehaibi et al., 2014; L. Huang et al., 2007; Lee et al., 2007, 2006; X. Li et al., 2016; Z. J. Liu et al., 2010; Lu et al., 2009; Shen & Ha, 2005; Sun, 2010; Tong et al., 2016, 2015; Wang et al., 2019; Wei et al., 2013; Yuan, 2008; Zafarmand et al., 2008). Of these, 10 case–control studies focused on rs1801282 C/G polymorphism (Bazina et al., 2015; L. Huang et al., 2007; Lee et al., 2007, 2006; X. Li et al., 2016; Shen & Ha, 2005; Tong et al., 2016, 2015; Wang et al., 2019; Zafarmand et al., 2008) and, seven case–control studies focused on rs3856806 C/T polymorphism (Chehaibi et al., 2014; Z. J. Liu et al., 2010; Lu et al., 2009; Sun, 2010; Wang et al., 2019; Wei et al., 2013; Yuan, 2008). In addition, 13 studies were based on Asian descendants, and three studies were based on European descendants. Four genotype methods were used among these studies, including polymerase chain reaction–restriction fragment length polymorphism (PCR‐RFLP), TaqMan, multilocus genotyping assay, and SNaPshot Multiplex Kit. All of the included characteristics are summarized in Table 1.

### 3.2 PPAR‐γ rs1801282 C/G polymorphism and IS risk

**FIGURE 2:**
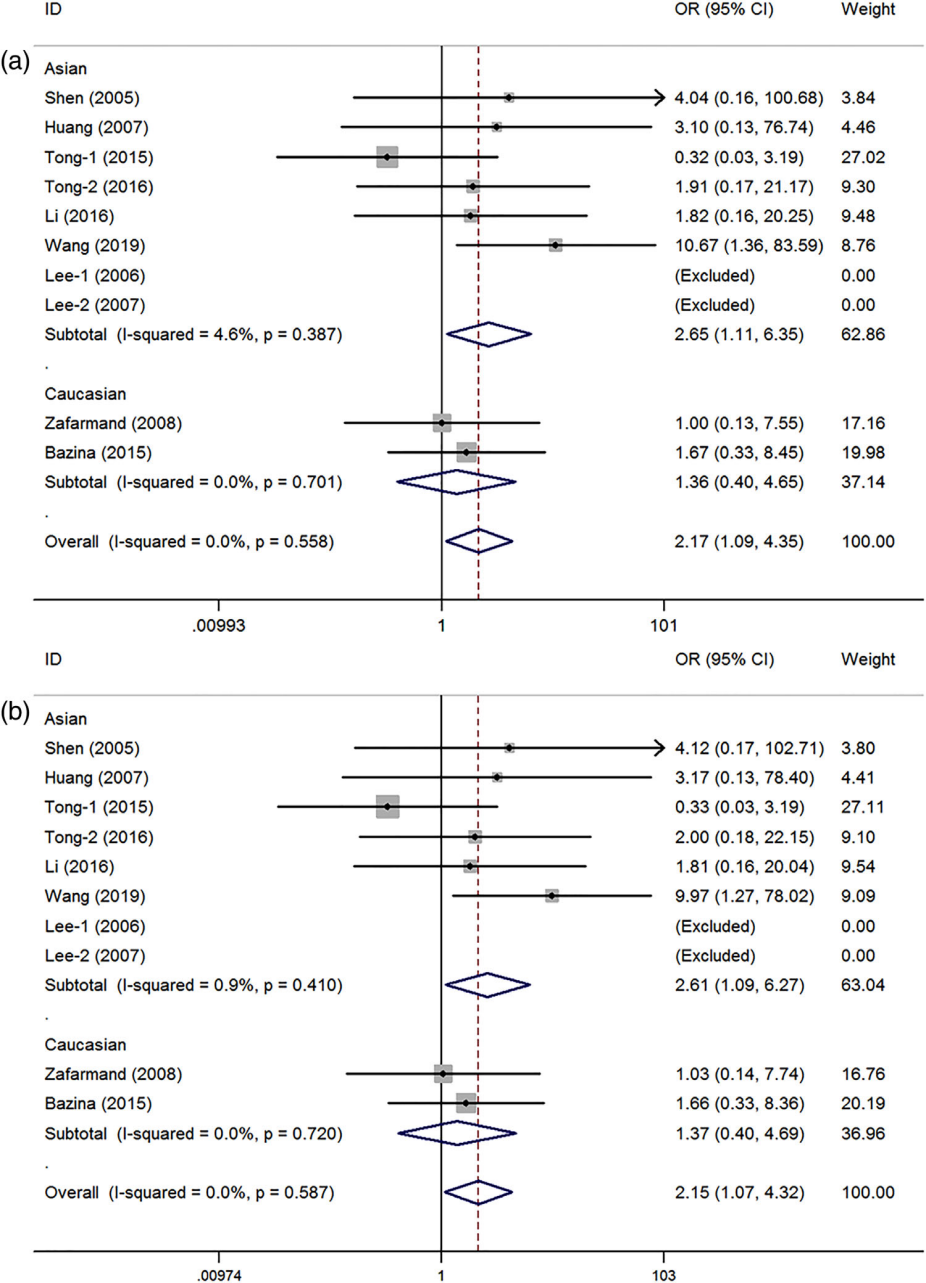
Odd ratios (OR) and 95% confidence interval (CIs) of the associations between peroxisome proliferator‐activated receptor‐γ (PPAR‐γ) rs1801282 C/G polymorphism and ischemic stroke susceptibility (a: GG vs. CC model; b: GG vs. CC+CG model)

**FIGURE 3:**
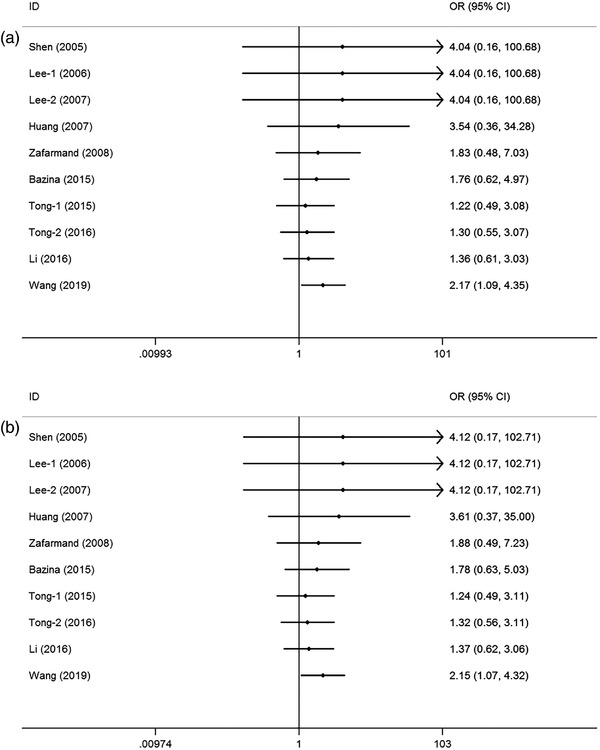
Cumulative meta‐analyses according to publication year on peroxisome proliferator‐activated receptor‐γ (PPAR‐γ) rs1801282 C/G polymorphism and ischemic stroke susceptibility (a: GG vs. CC model; b: GG vs. CC+CG model)

Ten case–control studies involving a total of 2804 patients and 4457 controls were identified for the PPAR‐γ2 rs1801282 C/G polymorphism and IS risk. The results of our meta‐analysis revealed a significant risk between the rs1801282 C/G polymorphism of PPAR‐γ and IS susceptibility in the general population (GG vs. CC: OR = 2.17 95%CI = 1.09–4.35, *p* = .03, *I* ^2 ^= 0%; GG vs. CC+CG: OR = 2.15, 95%CI = 1.07–4.32, *p* = .03, *I* ^2 ^= 0%) (Table 2, Figure 2a for GG vs. CC model; Figure 3b for GG vs. CC+CG model). Stratification analysis indicated an increased IS risk with the C allele in the Asian population (GG vs. CC: OR = 2.65, 95%CI = 1.11–6.35, *p* = .03, *I* ^2 ^= 4.6%; GG vs. CC+CG: OR = 2.61, 95%CI = 1.09–6.27, *p* = .03, *I* ^2 ^= 0%) (Table 2). Moreover, significant associations between the rs1801282 C/G polymorphism and IS risk were also found in the subgroups with population‐based (PB) controls (GG vs. CC: OR = 2.21, 95%CI = 1.07–4.55, *p* = .03, *I* ^2 ^= 0%; GG vs. CC+CG: OR = 2.19, 95%CI = 1.06–4.53, *p* = .03, *I* ^2 ^= 0%) and among those studies with more than 500 individuals (GG vs. CC: OR = 2.26, 95%CI = 1.26–8.43, *p* = .02, *I* ^2 ^= 0%; GG vs. CC+CG: OR = 3.22, 95%CI = 1.24–8.38, *p* = .02, *I* ^2 ^= 0%) (Table 2).

**TABLE 2:** Summary odd ratios (ORs) and 95% confidence interval (CI) of peroxisome proliferator‐activated receptor‐γ (PPAR‐γ) polymorphism and ischemic stroke risk

		Allelic model: G vs. C				Codominant model: CG vs. CC				Codominant model: GG vs. CC				Dominant model: CG+GG vs. CC				Recessive model: GG vs. CC+CG			
Rs1801282C/G	*N* *	OR	95% CI	*p*	*I* ^2^(%)	OR	95% CI	*p*	*I* ^2^(%)	OR	95% CI	*p*	*I* ^2^(%)	OR	95% CI	*p*	*I* ^2^(%)	OR	95% CI	*p*	*I* ^2^(%)
Total	10	0.90	0.62–1.29	.55	76.5	0.84	0.58–1.21	.35	73.2	2.17	1.09–4.35	.03	0	0.86	0.59–1.26	.44	75.4	2.15	1.07–4.32	.03	0
Ethnicity																					
Asian	8	0.86	0.54–1.37	.52	81.6	0.80	0.50–1.28	.35	79.1	2.65	1.11–6.35	.03	4.6	0.83	0.52–1.33	.43	80.7	2.61	1.09–6.27	.03	0.9
Caucasian	2	1.02	0.70–1.48	.92	0	0.96	0.62–1.48	.86	0	1.36 6	0.40–4.65	.62	0	0.99	0.65–1.50	.96	0	1.37	0.40–4.69	.61	0
Control design																					
PB	8	0.95	0.64–1.41	.81	76.2	0.89	0.59–1.33	.56	72.7	2.21	1.07–4.55	.03	0	0.92	0.61–1.39	.68	75.0	2.19	1.06–4.53	.03	0
HB	2	0.71	0.25–2.00	.51	83.1	0.68	0.24–1.89	.46	81.0	1.82	0.16–20.25	.62	NA	0.69	0.24–1.97	.49	82.4	1.81	0.16–20.04	.63	NA
Subjects number																					
<500	5	0.91	0.68–1.22	.53	0	0.85	0.61–1.18	.34	0	1.29	0.45–3.70	.63	0	0.87	0.64–1.21	.41	0	1.30	0.45–3.70	.63	0
≥500	5	0.91	0.50–1.66	.75	88.3	0.85	0.46–1.57	.61	86.8	3.26	1.26–8.43	.02	0	0.88	0.47–1.64	.69	87.9	3.22	1.24–8.38	.02	0
rs3856806 C/T		T vs. C				CT vs. CC				TT vs. CC				CT+TT vs. CC				TT vs. CC+CT			
Total	7	0.87	0.59–1.28	.48	88.7	0.88	0.59–1.29	.50	81.7	1.00	0.45–2.22	1.00	78.8	0.86	0.56–1.32	.49	86.7	1.03	0.51–2.08	.94	72.9
HWE status																					
HWE‐yes	5	1.00	0.66–1.52	.99	87.5	0.95	0.59–1.53	.83	85.3	1.46	0.66–3.21	.35	61.7	0.98	0.59–1.61	.92	87.6	1.54	0.82–2.89	.18	43.2
HWE‐no	2	0.60	0.45–0.80	<.01	0	0.69	0.47–1.03	.07	0	0.46 6–0.	0.26–0.82	.01	0	0.61	0.43–0.87	.01	0	0.50	0.28–0.88	.02	0
Ethnicity																					
Asian	6	0.94	0.64–1.39	.76	86.6	0.91	0.59–1.40	.66	83.1	1.23	0.58–2.63	.59	66.4	0.92	0.58–1.45	.72	86.1	1.29	0.68–2.43	.43	53.0
Subjects number																					
<500	6	0.79	0.52–1.18	.25	83.2	0.80	0.50–1.27	.34	79.2	0.75	0.40–1.39	.36	44.4	0.77	0.48–1.25	.30	82.6	0.73	0.48–1.09	.13	20.5

*Note: I* ^2^ for Heterogeneity test.

Abbreviations: HB, hospital‐based; HWE, Hardy–Weinberg equilibrium; control; PB, population‐based.

* Numbers of comparisons.

**FIGURE 4:**
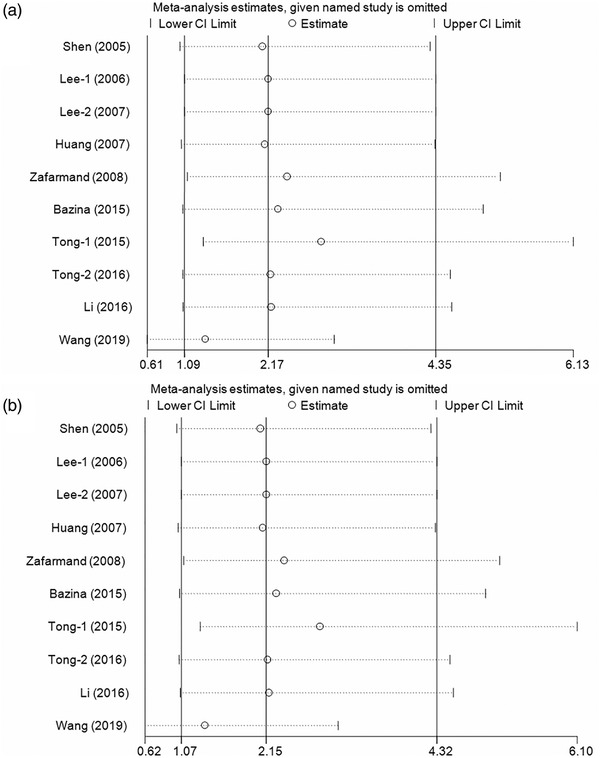
Sensitivity analysis when each study was removed with publication year on peroxisome proliferator‐activated receptor‐γ (PPAR‐γ) rs1801282 C/G polymorphism and ischemic stroke susceptibility (a: GG vs. CC model; b: GG vs. CC+CG model)

**FIGURE 5:**
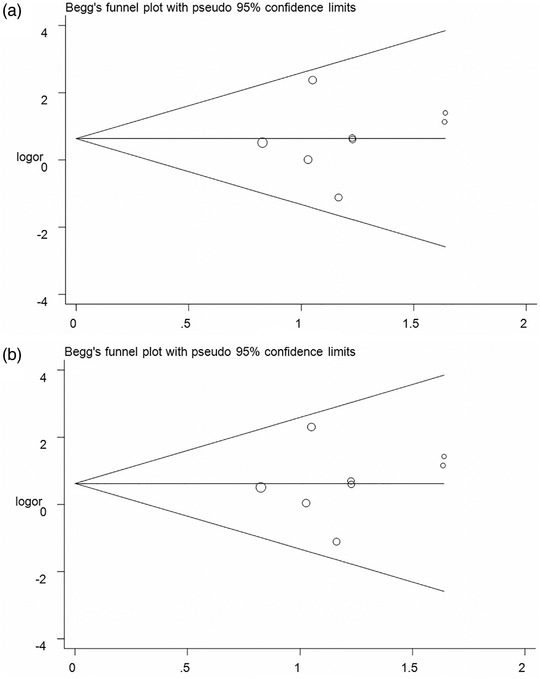
Funnel plot analysis for detect publication bias on peroxisome proliferator‐activated receptor‐γ (PPAR‐γ) rs1801282 C/G polymorphism and ischemic stroke susceptibility (a: GG vs. CC model; b: GG vs. CC+CG model). Circles represent the weight of the studies

Cumulative analyses presented a positive result with the studies added to the homozygous and recessive models (Figure 3a for GG vs. CC model; Figure 3b for GG vs. CC+CG model). Sensitivity analyses indicated that the results fluctuated slightly only when the study by Tong et al. (2015) was removed (Figure 4a for GG vs. CC model; Figure 4b for GG vs. CC+CG model). In addition, no significant asymmetrical funnel plot was observed for publication bias among the included studies, which was further verified by Egger's test (G vs. C: *p* = .08; CG vs. CC: *p* = .09; GG vs. CC: *p* = .77; CG+GG vs. CC: *p* = .09; GG vs. CC+CG: *p* = .74) (Figure 5a for GG vs. CC model; Figure 5b for GG vs. CC+CG model).

### 3.3 PPAR‐γ rs3856806 C/T polymorphism and IS risk

Seven case–control studies, involving 1873 patients and 1769 controls, were identified for the meta‐analysis of the association between PPAR‐γ rs3856806 C/T polymorphism and IS risk. Our results suggest that there is no significant association between the rs3856806 C/T polymorphism and IS risk (T vs. C: OR = 0.87, 95%CI = 0.59–1.28, *p* = .48, *I* ^2 ^= 88.7%; CT vs. CC: OR = 0.50, 95%CI = 0.59–1.29, *p* = .50, *I* ^2 ^= 81.7%; TT vs. CC: OR = 1.00, 95%CI = 0.45–2.20, *p* = 1.00, *I* ^2 ^= 78.8%; CT+TT vs. CC: OR = 0.86, 95%CI = 0.56–1.32, *p* = .49, *I* ^2 ^= 86.7%; TT vs. CC+CT: OR = 1.03, 95%CI = 0.51–2.08, *p* = .94, *I* ^2 ^= 72.9%) (Table 2). Moreover, the synthesized result demonstrated a null association between the rs3856806 C/T polymorphism and IS risk in the subgroup analysis.

Cumulative analyses and sensitivity analyses were conducted, and the results indicated a consisted trend without any fluctuation. In addition, no publication bias was observed with symmetrical funnel plots in all five genetic models, which was further verified with the Egger's test (T vs. C: *p* = .17; CT vs. CC: *p* = .30; TT vs. CC: *p* = .16; CT+TT vs. CC: *p* = .26; TT vs. CC+CT: *p* = .11).

## 4 DISCUSSION

According to the World Health Organization (WHO), stroke is one of the leading causes of mortality and disability worldwide and is responsible for approximately 6.1million deaths in 2019 (World Health Organization, 2020). IS frequently results from the occlusion of cerebral blood vessels, usually caused by thrombosis with blood clot formation or embolism with intracranial stenosis, accounting for approximately 85% of all stroke incidences (Zhou et al., 2019). With an increase in the aging population, the number of IS patients has been increasing in recent years and is predicted to continue, which brings a substantial economic and social burden to the public health system.

Hypertension, hyperlipidemia, hyperglycemia, atherosclerosis, and arterial stenosis have been demonstrated to be the main causes of IS. In addition, smoking, drinking, unhealthy lifestyle habits, and psychosocial stress have additionally been suggested as high‐risk factors for the development of IS. Thrombosis caused by atherosclerosis and inflammatory injury of the vascular endothelium is one of the most direct causes of IS and is significantly associated with aberrant expression or dysfunction of the PPAR family. PPAR‐γ is a member of the nuclear receptor superfamily and a ligand‐activated transcription factor which is widely expressed in adipose cells and tissues and plays a critical role in the regulation of adipogenesis metabolism, insulin sensitivity, energy balance, inflammation, angiogenesis microvascular lesions, and atherosclerosis (Rocha et al., 2015).

In the past decades, PPAR‐γ agonists such as cyclooxygenase‐2, interleukin‐1β (IL‐1β), and tumor necrosis factor‐α, which are known to decrease the level of inflammation in many IS models, have been shown to attenuate proinflammatory mediators during IS development. In contrast, murine stroke models with PPAR‐γ knocked out also present a stronger proinflammatory response with a higher risk of secondary intracerebral hemorrhage (Gliem et al., 2015). Moreover, other studies have shown that pioglitazone decreases the expression of inflammatory cytokines IL‐6, IL‐1β, and monocyte chemoattractant factor‐1 and reduces reperfusion injury of the intracranial artery by diminishing the infiltration of M1 macrophages into the cerebral artery. In contrast, the PPAR‐γ antagonist GW9662 reversed this protective function, further supporting the anti‐inflammatory effect of PPAR‐γ (Yang et al., 2020). In addition, PPAR‐γ agonists have been suggested to reduce the risk of recurrent stroke and total events of cardiovascular death or stroke by protecting against arteriosclerosis formation and maintaining the stability of carotid plaques (J. Liu & Wang, 2019; Marfella et al., 2006). Modrick et al. (2012) suggested that PPAR‐γ might play a protective role in vascular aging by protecting against age‐induced oxidative stress and endothelial dysfunction. Moreover, PPAR‐γ activation is known to enhance angiogenesis and the migration of human brain microvascular endothelial cells through the formation of the fibroblast growth factor‐21/fibroblast growth factor receptor‐1/β‐Klotho complex, and improve the ability of vascular recanalization and nerve repair after stroke (W. Huang et al., 2019). All these lines of evidence demonstrate that PPAR‐γ plays an important role in the pathogenesis of IS. However, the underlying pathogenesis of PPAR‐γ in the IS remains unclear.

SNPs are one of the most prevalent forms of gene mutations which can influence gene expression and protein activity. In terms of the PPAR‐γ gene, rs1801282 C/G and rs3856806 C/T polymorphisms are the most common SNP loci, which have been reported to be involved in various disease susceptibilities in the past decades. In 1997, the rs1801282 C/G polymorphism locus was first reported by Yen et al. (1997). This mutation comprises an exchange of cytosine for guanine, resulting in the substitution of alanine for proline at codon 12 of exon B. The Ala allele reduces the binding affinity to the cognate promoter element as well as promoter activity and transcription of PPAR‐γ (Deeb et al., 1998). In 2005, Shen and Ha (2005) conducted the first case–control study focusing on the PPAR‐γ gene rs1801282 C/G polymorphism and IS risk in a Chinese population; however, they did not find any significant association between this mutation and IS. Since then, subsequent series of studies have been conducted, and some inconsistent or contradictory results have been reported. Lee et al. (2006) found a potential protective trend of the rs1801282 C/G genotype in IS patients with type 2 diabetes in Korean people (OR = 0.43, *p* = .025). Tong et al. (2016) additionally reported a protective effect against IS formation in a Chinese population. In contrast, another study by Wang et al. (2019) demonstrated that the G allele presented a significantly higher frequency of the rs1801282 polymorphism than that in the control group and carried a 1.844‐fold increased risk of IS (OR = 1.844, *p* < .001). However, most studies have suggested that there is no significant correlation between this mutation and IS risk (Bazina et al., 2015; L. Huang et al., 2007; Zafarmand et al., 2008). Ethnic differences, small sample sizes of studies, and differences in scientific research approaches and qualities of data may be the main reasons for the inconsistent conclusions among previous studies.

Meta‐analysis is a useful statistical method that combines quantitative methods with synthetic data from published studies and draws conclusions on the same theme. For the PPAR‐γ gene, the rs1801282 C/G polymorphism could result in a missense mutation and lead to an amino acid change from proline to alanine. This alteration could reduce the transcriptional activity and regulate the lipoprotein lipase activity, thereby affecting the removal of triglycerides, which leads to atherosclerosis due to dyslipidemia (Schneider et al., 2002). Therefore, we conducted this meta‐analysis with 10 case–control studies to assess the precise association between the PPAR‐γ rs1801282 polymorphism and IS susceptibility. Overall, the synthesized data suggested that there were significant associations between the rs1801282 polymorphism and IS susceptibility in the homozygous and recessive models. Additionally, ethnic stratification indicated that there was a significant increase in IS risk in Asians with the above‐mentioned genotypes. These results indicate that the polymorphism locus may play an important role in the occurrence of IS, along with other environmental factors. Furthermore, the differences in ethnicity may be a positive factor contributing to the increased risk in Asians, suggesting that this rs1801282 polymorphism could be involved in IS susceptibility in specific races.

In terms of the PPAR‐γ gene rs3856806 C/T polymorphism, this mutation is located in exon 6 and results in a substitution of cytosine for thymine, which causes a synonymous mutation with histidine. A previous study reported that although this polymorphism was a silent mutation locus, it still presented a protective effect on atherosclerotic lipid profiles in various cardiovascular diseases. Chehaibi et al. (2014) and other researchers found a significant reduction in serum triglyceride and apolipoprotein B levels in T allele carriers in IS patients. In addition, Matsunaga et al. (2020) also reported that participants with the TT genotype of C/T polymorphism presented with a 45% lower risk of high low‐density lipoprotein cholesterol and a 42% lower risk of dyslipidemia. This evidence indicates that rs3856806 C/T reduces the risk of hyperlipidemia and further diminishes the risk of atherosclerotic vessels and IS in patients with type 2 diabetes mellitus. However, the exact role of the rs3856806 C/T polymorphism in IS risk was not consistent in published articles. Lu et al. and others believed that the T allele of the rs3856806 C/T polymorphism had a protective effect against IS occurrence (Z. J. Liu et al., 2010; Lu et al., 2009; Wei et al., 2013). In contrast, Yuan (2008) and Wang et al. (2019) observed an increased risk of IS in individuals with T polymorphism. However, the remaining studies showed that there was no clear correlation between this polymorphism and IS risk (Sun, 2010; Wei et al., 2013). In the current meta‐analysis, we found no statistically significant association between this polymorphism and IS risk in the general analysis. Moreover, a similar result of no association was observed in the ethnic and other subgroups. The current results indicate that the single‐site mutation is not sufficient to affect the susceptibility to IS.

To our knowledge, this study is the first meta‐analysis to focus on PPAR‐γ gene polymorphisms (rs1801282 C/G and rs3856806 C/T) and IS risk. Some advantages exist in this study that help to improve the stability and credibility of the results: (1) a comprehensive and scientific retrieval strategy was used to collect complete information; (2) the current study included a large sample size with all published studies to date; (3) rigorous and accurate methods, including the NOS evaluation, HWE test, cumulative/sensitivity analyses, and publication bias were used one at a time; and (4) subgroup analyses in two polymorphism loci were conducted to explore potential associations. Inevitably, there are certain limitations that need to be addressed: (1) only two polymorphism loci were examined in this study separately, and the interaction mechanism between loci and heterozygous/homozygous state was not involved; (2) we incorporated studies that mainly originated from Asian and European countries, and the number of collected studies or participants from each study was insufficient, which may lead to uncertainty and variations in results; (3) This study was based on unadjusted data, and other environmental exposure factors, such as serum triglyceride, blood pressure, and body mass index were not involved, which limits our understanding of the underlying mechanisms; and (4) some moderate heterogeneity was observed in genetic models among two polymorphism loci, which might affect the reliability and accuracy of the results.

## 5 CONCLUSION

In summary, this meta‐analysis suggests that the PPAR‐γ rs1801282 C/G polymorphism may be associated with IS risk in Asians. More studies with larger sample sizes in different ethnic backgrounds are needed to either refute or verify the current conclusions in the future.

## CONFLICT OF INTEREST

The authors declare no conflict of interest.

## AUTHOR CONTRIBUTIONS

Fan Cheng, Xiao‐Min Si, and Lan Zhou conceived the study. Fan Cheng and Xiao‐Min Si searched the databases and extracted the data. Xiao‐Min Si and Gong‐Li Yang analyzed the data. Fan Cheng, Xiao‐Min Si, and Gong‐Li Yang wrote the draft of the paper. Lan Zhou reviewed the proof. All of the authors approved the final manuscript.

### PEER REVIEW

The peer review history for this article is available at https://publons.com/publon/10.1002/brb3.2434

## Data Availability

All data collected or analyzed for this study are available from the corresponding author upon reasonable request to any researchers.
